# Novel Epigenetics Control (EpC) Nanocarrier for Cancer Therapy Through Dual-Targeting Approach to DNA Methyltransferase and Ten-Eleven Translocation Enzymes

**DOI:** 10.3390/epigenomes9010006

**Published:** 2025-02-11

**Authors:** Risa Mitsuhashi, Kiyoshi Sato, Hiroyoshi Kawakami

**Affiliations:** Department of Applied Chemistry for Environment, Graduate School of Urban Environmental Sciences, Tokyo Metropolitan University, 1-1 Minami-Osawa, Hachioji 192-0397, Tokyo, Japan

**Keywords:** DNA methylation, TET enzymes, 5-aza-2′-deoxycytidine (5-aza-dC), epigenetics control (EpC) nanocarrier, cell cycle arrest

## Abstract

Background/Objectives: Aberrant hypermethylation in the promoter regions of tumor suppressor genes facilitates the pathogenesis and progression of cancer. Therefore, inhibitors targeting DNA methyltransferase (DNMT) have been tested in clinical studies. However, the current monotherapy of DNMT inhibitors shows limited efficacy. Furthermore, the mechanism of action of DNMT inhibitors is DNA replication-dependent. To address these limitations, we developed a novel core–shell-type “epigenetics control (EpC) nanocarrier” that encapsulated decitabine (5-aza-dC) in the PLGA core nanoparticle and hybridized TET1 gene-encoding pDNA on the lipid shell surface. This study aimed to evaluate whether the dual delivery of DNMT inhibitors and pDNA of TET1 could synergistically enhance tumor suppressor gene expression and induce cell cycle arrest and/or apoptosis in cancer cells. Herein, we demonstrate the potential of the EpC carrier in HCT116 human colon cancer cells to upregulate tumor suppressor gene expression and rapidly achieve cell cycle arrest. Methods: PLGA core nanoparticles were prepared by the W/O/W double emulsion method. The formation of core–shell nanoparticles and complexation with pDNA were investigated and optimized by dynamic light scattering, zeta potential measurement, and agarose gel electrophoresis. The cellular uptake and transfection efficiency were measured by confocal laser scanning microscopy and a luciferase assay, respectively. The expression of p53 protein was detected by Western blotting. The anti-tumor effects of the EpC nanocarrier were evaluated by cell cycle analysis and an apoptosis assay. Results: The EpC nanocarrier delivered the DNMT inhibitor and TET gene-encoding pDNA into HCT116 cells. It promoted the expression of the tumor suppressor protein p53 and induced rapid cell cycle arrest in the G2/M phase in HCT116 cells. Conclusions: Our findings suggest that the dual-targeting of DNMT and TET enzymes effectively repairs aberrant DNA methylation and induces growth arrest in cancer cells, and the dual-targeting strategy may contribute to the advancement of epigenetic cancer therapy.

## 1. Introduction

Cancer is caused not only by the accumulation of genetic abnormalities and mutations but also by the alternation of epigenetic modifications, such as aberrant DNA methylations and histone acetylations/methylations [1,2]. In cancer cells, the promoter regions are hypermethylated, while other regions are hypomethylated [3]. The methylation and demethylation of cytosines located in the promoter regions (CpG islands) strongly influence the binding of transcription factors to DNA. Thus, these epigenetic alternations regulate gene expression in cancer cells. For example, hypermethylation in CpG islands of the tumor suppressor gene TP53 silences the expression of p53 protein, leading to both the development and progression of cancer [4,5]. Therefore, the downregulation of the DNA methylation of tumor suppressor genes is a promising approach in cancer treatment.

Epigenetic therapies can potentially reverse epigenetic changes and restore gene expression affected by the alternations. Accordingly, restoring aberrant epigenetic modifications using inhibitors can be a suitable therapeutic approach in cancers. Currently, clinical research on DNA methyltransferase (DNMT) inhibitors [6,7] and histone deacetylase (HDAC) inhibitors [8,9] has been conducted in cancer therapy. The two FDA-approved DNMT inhibitors, 5-azacytidine and decitabine (5-aza-dC), and four approved HDAC inhibitors, vorinostat, romidepsin, panobinostat, and belinostat, have mainly been used in clinical trials and have demonstrated anti-tumor potential towards various cancers [10]. However, single epi-drug treatments’ limited efficacy and susceptibility to side effects and drug resistance represent the primary issues with their use [11]. In addition, the action of cytidine analog DNMT inhibitors is a DNA replication-dependent “passive” demethylation mechanism [12]. Therefore, it takes a relatively longer time to observe efficacy compared with the fast response of HDAC inhibitors. Moreover, paradoxically, the dilution effect of 5mC does not occur unless the cancer cells continue to proliferate. Hence, epigenetic therapy targeting aberrant DNA methylation in cancer requires a different strategy.

Although DNA methylation is a tightly regulated epigenetic modification and generally stable compared with reversible histone modifications, recent studies have revealed ten-eleven translocation (TET) enzymes that act as erasers for DNA methylation to catalyze the stepwise oxidation of 5-methylcytosine (5mC) to 5-hydroxymethylcyctosine (5hmC), 5-formylcytosine (5fC), and 5-carboxylcytosine (5acC), which has been found to facilitate the DNA replication-independent “active” demethylation process [12]. However, low expression or loss of function in TET enzymes leads to tumorigenesis and malignancy [13,14]. Therefore, TET enzymes have emerged as a novel target for epigenetic therapy, and the activation or upregulation of TET enzymes is expected in the rapid treatment of aberrant DNA methylation.

Previously, we reported core–shell-type nanoparticles as “epigenetics control (EpC) nanocarriers” for the treatment of COPD through the combination of ROS scavenger with HDAC2 gene-encoding plasmid DNA (pDNA) [15] and for the suppression of cancer EMT through the combination of a mitochondria-targeting anti-oxidant with HAT1-encoding pDNA [16], respectively. In this study, we designed a novel type of EpC nanocarrier to improve the response of epi-drugs towards aberrant DNA methylation by simultaneously delivering a DNMT inhibitor and the pDNA of the TET gene into cancer cells using a core–shell-type EpC nanocarrier strategy. The brand new EpC nanocarrier that encapsulates 5-aza-dC in PLGA core nanoparticle and hybridizes TET1-encoding pDNA on DOTMA/DOPE lipid shell can ameliorate the aberrant DNA methylation and promote the expression of the tumor suppressor p53 gene, leading to fast cell cycle arrest in human HCT116 colorectal carcinoma cells.

## 2. Results

### 2.1. Preparation of EpC Nanocarrier

**Figure 1:**
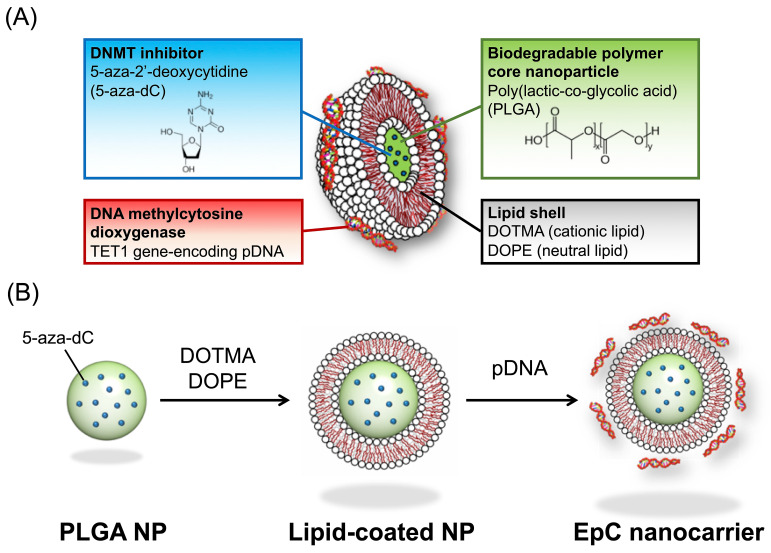
Structure (**A**) and preparation scheme (**B**) of the novel EpC nanocarrier for dual-targeting DNMT and TET1 enzymes in colorectal cancer cells.

The structure of the EpC nanocarrier is shown in Figure 1. The core of the EpC nanocarrier comprised an FDA-approved biodegradable PLGA nanoparticle, in which a DNMT inhibitor (5-aza-dC) was loaded for sustained release. The surface of the PLGA nanoparticles was coated with a lipid bilayer of a cationic lipid (DOTMA) and a neutral lipid (DOPE). Furthermore, TET1 gene-encoding pDNA was hybridized on the cationic surface of the lipid-coated PLGA nanoparticles by electrostatic interactions.

**Figure 2:**
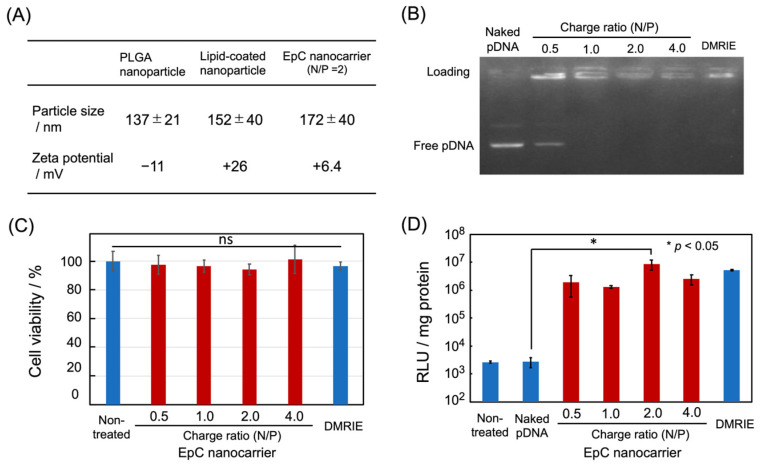
Characterization of EpC nanocarrier. (**A**) Mean particle size and zeta potential of nanoparticles measured by DLS. (**B**) pDNA loading ability of lipid-coated PLGA nanoparticles analyzed by agarose gel electrophoresis. (**C**) Cytotoxicity of EpC nanocarrier for WI-38 cells. (**D**) Transfection efficiency of EpC nanocarrier in HCT116 cells verified by luciferase assay.

The preparation of the EpC nanocarrier was as follows. First, PLGA core nanoparticles were formed by the W/O/W double-emulsion method [17]. Hydrophobic drugs can be encapsulated into PLGA particles by the O/W emulsion method, but 5-aza-dC is a hydrophilic drug. Thus, the W/O/W double-emulsion method was used in the present study. A W/O emulsion was formed by the sonication of the mixture of an aqueous drug solution (internal aqueous phase) and a dichloromethane/methanol solution of PLGA(dispersed phase). The W/O emulsion was dispersed in an aqueous PVA (polyvinyl alcohol) solution (external aqueous phase), followed by sonication to form a W/O/W emulsion. Then, the dichloromethane/methanol was evaporated to precipitate PLGA nanoparticles that retained the internal aqueous phase in which the drug was dissolved. Finally, the dispersion of PLGA nanoparticles was freeze-dried. The drug encapsulation rate was 27.1%, calculated from the amount of the drug used versus the amount of the drug encapsulated in PLGA nanoparticles, determined by the absorbance of 5-aza-dC. The size and zeta potential of re-dispersed PLGA nanoparticles were ca. 137 nm and −11 mV, respectively, determined by dynamic light scattering (DLS) measurements (Figure 2A).

Next, the surface of the PLGA nanoparticles was coated with a lipid bilayer by the sonication of a PLGA nanoparticle dispersion with a thin film made from a 1:1 mixture of the cationic lipid (DOTMA) and the neutral lipid (DOPE). The mean particle size increased by ca.15 nm, and the zeta potential was changed from a negative value to a positive value, respectively, suggesting the formation of core–shell-type lipid-coated PLGA nanoparticles (Figure 2A).

The pDNA loading ability of the lipid-coated PLGA nanoparticles was investigated by agarose gel electrophoresis at various nitrogen/phosphorus (N/P) charge ratios (Figure 2B). The free pDNA band was observed at the N/P ratio of 0.5, whereas the band completely disappeared above 1, indicating the complex formation between lipid-coated PLGA nanoparticles and pDNA. The hybridization was also confirmed by DLS measurements (Figure 2A). After forming the pDNA complex (at N/P = 2), the particle size increased by ca.15 nm, and the zeta potential decreased, suggesting the formation of the EpC nanocarrier shown in Figure 1. The particle size of the EpC nanocarrier (200 nm or less) is suitable for cellular uptake, and the positive charge of the EpC nanocarrier can contribute to intracellular uptake.

To evaluate the cytotoxicity of the EpC nanocarrier, the nanocarrier was added to human fetal lung fibroblast normal cells (WI-38 cells). An Alamar Blue assay showed no significant difference in the cell viability at any charge ratio compared to untreated cells, suggesting the absence of cytotoxicity (Figure 2C).

Finally, the gene expression efficiency of the pDNA hybridized on the EpC nanocarrier was evaluated by transfection experiments using luciferase as a reporter gene. The EpC nanocarriers hybridized with luciferase gene-encoding pDNA were added to colon cancer HCT116 cells, and the transfection activity was evaluated by a conventional luciferase assay. The results showed significant gene expression at all charge ratios compared to untreated cells and the addition of naked pDNA, but the highest gene expression was observed at a charge ratio of N/P = 2 (Figure 2D).

From these observations, the charge ratio of N/P = 2 was used for the following experiments.

### 2.2. Cellular Uptake of EpC Nanocarrier

**Figure 3:**
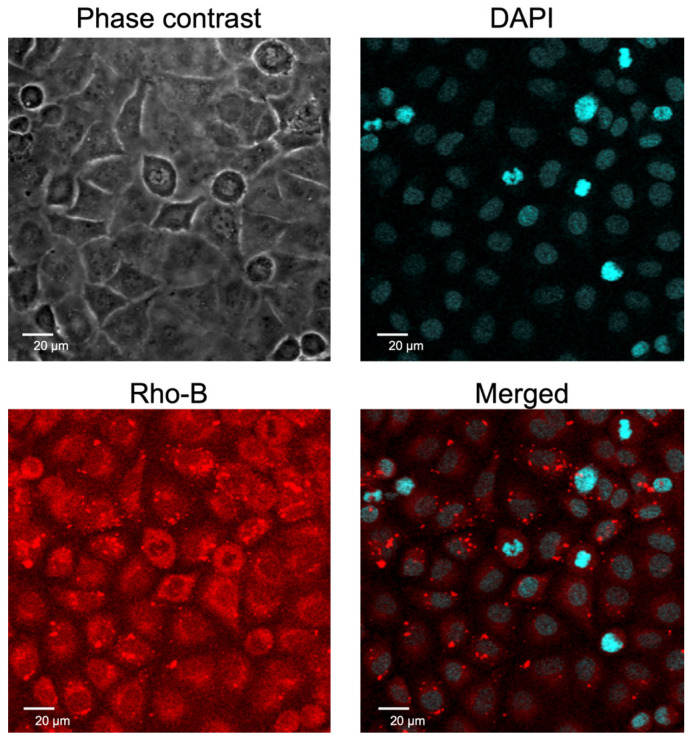
Cellular uptake of Rho-B-encapsulated EpC nanocarrier into HCT116 cells observed by confocal laser scanning microscopy. The cells were stained with DAPI after incubation with EpC nanocarrier.

Next, the cellular uptake of the EpC nanocarrier into HCT116 cells and the intracellular distribution of the encapsulated molecules in the PLGA core were assessed by confocal laser scanning microscopy (CLSM) using a fluorescent dye (Rho-B, as an alternative to DNMT inhibitor)-encapsulated EpC nanocarrier. The CLSM images of nanocarrier-treated cells stained with DAPI are shown in Figure 3. The blue fluorescence of DAPI indicates the location of the nucleus, and the red fluorescence of Rho-B shows the location of the core-encapsulated molecules. The merged image shows that the fluorescence of the two dyes overlapped in the nuclei, suggesting that the EpC nanocarrier was taken up into the cells, and the encapsulated molecules were delivered into the nuclei of HCT116 cells.

From the characterization of the nanoparticles, we successfully prepared the EpC nanocarrier and found that the highest gene expression was observed at a charge ratio of N/P = 2. In addition, toxicity evaluation showed no change in the cell viability of normal WI-38 cells. Furthermore, the observation of intracellular localization suggested that the DNMT inhibitor would be transferred to the nuclei of cancer cells.

### 2.3. Upregulation of p53 Protein in HCT116 Cells

**Figure 4:**
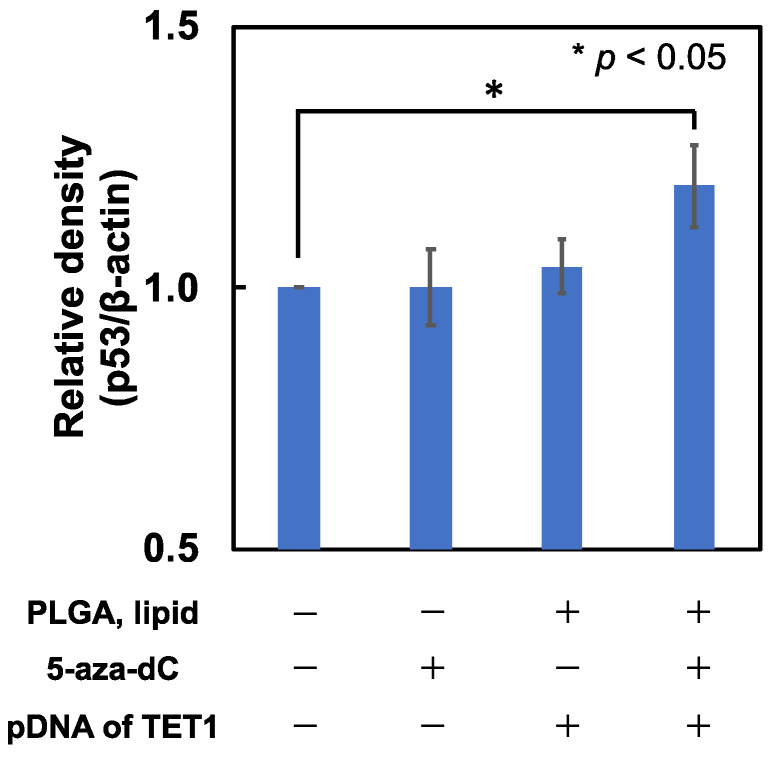
Expression of p53 protein in HCT116 cells with various treatments analyzed by Western blotting.

The ability of the EpC nanocarrier to induce tumor suppressor gene TP53 in HCT116 cells was assessed by measuring the p53 protein expression level using Western blotting (Figure 4). Compared with non-treated HCT116 cells, the expression of p53 protein in the EpC nanocarrier-treated cells increased, indicating the induction of TP53 gene expression by the EpC nanocarrier. On the other hand, single-drug treatments with 5-aza-dC or TET1 gene-encoding pDNA hybridized drug-free nanocarrier showed no significant increase in the expression of p53 protein. Thus, the co-delivery of the DNMT inhibitor and the pDNA of TET1 using the EpC nanocarrier was found to be an effective approach to the recovery of p53 expression in HCT116 cells, suggesting that the hypermethylation in the TP53 gene promoter region was reduced by the combined treatment.

### 2.4. Anti-Tumor Effects of EpC Nanocarrier

**Figure 5:**
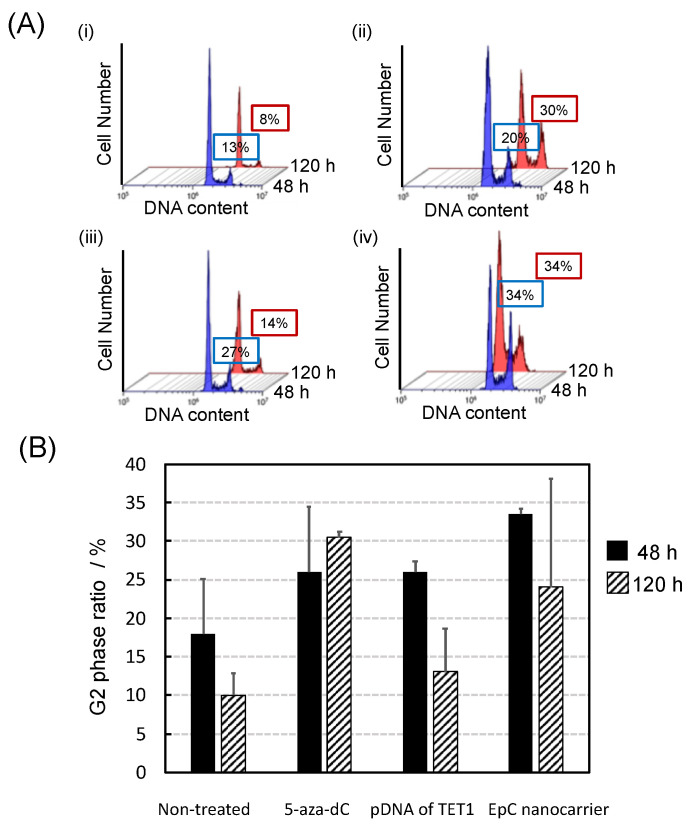
(**A**) A flow cytometry cell cycle analysis in HCT116 cells. (**i**) NT, (**ii**) 5-aza-dC, (**iii**) pDNA of TET1, and (**iv**) EpC nanocarrier. (**B**) The ratio of G2/M cell cycle arrest cells.

Next, the cell proliferation inhibitory effect of the EpC nanocarrier on HCT116 cells was evaluated by cell cycle analysis using flow cytometry. The time course of the G2/M phase ratio after treatments showed that G2/M cell cycle arrest was efficiently induced and maintained by the EpC nanocarrier treatment (Figure 5). By 48 h of treatment, the average of G2/M ratio was 26% for 5-aza-dC and 26% for the pDNA of TET1, suggesting that the TET1 enzyme plays a major role in the DNA demethylation process in the early stage. With EpC nanocarrier treatment, the average G2/M ratio increased to 33%. Taking account of the percentage of G2/M cell-cycle-arrested cells in control being 18%, the effect of the EpC nanocarrier was the sum of the effects of 5-aza-dC and TET1. After 120 h of treatment, the average G2/M ratio had increased to 30% for 5-aza-dC, whereas it had significantly decreased to 13% for the pDNA of TET1, suggesting that the controlled release of 5-aza-dC from the PLGA core nanoparticles contributed to the sustained efficacy. This effect was also observed for the EpC nanocarrier. The average G2/M ratio remained at 24%, suggesting that the EpC nanocarriers could efficiently control the DNA methylation/demethylation process from the early to later stages.

**Figure 6:**
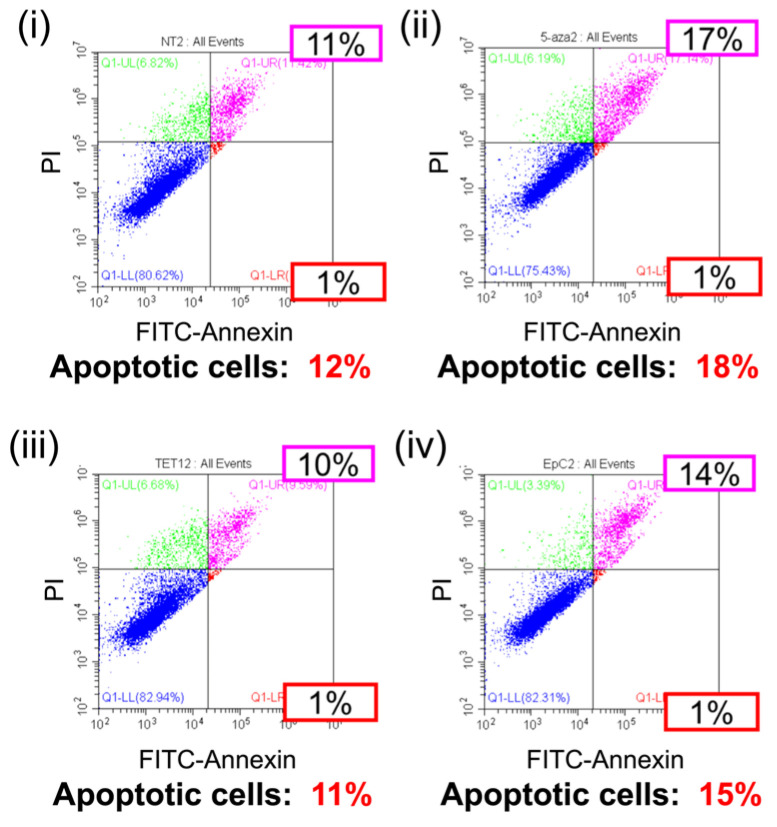
Flow cytometry analysis of the apoptosis in HCT116 cells. (**i**) NT, (**ii**) 5-aza-dC, (**iii**) TET1 carrier, and (**iv**) EpC nanocarrier.

The overexpression of wild-type p53 in cancer cells leads to growth arrest or apoptosis in cancer cells [18]. Therefore, we evaluated the apoptosis of EpC nanocarrier-treated HCT116 cells. The results of the annexin V assay are shown in Figure 6 and Figure S2. Apoptosis occurred in 11–12% of non-treated cells, 16–18% of cells treated with 5-aza-dC, 10–11% of cells treated with TET1-encoding pDNA, and 14–15% of cells treated with EpC nanocarrier. EpC nanocarrier treatment and single-drug treatment with the DNMT inhibitor showed a slight increase in apoptosis cells, but there were no significant differences between the treatments.

These results suggest that the EpC nanocarrier may repair the aberrant DNA methylation in cancer cells by co-delivering TET1 gene-encoding pDNA and 5-aza-dC and suppress cancer cell proliferation by promoting G2/M cell cycle arrest via p53 protein expression.

## 3. Discussion

Although the pivotal roles of TET enzymes in cancer development and progression have been established by recent investigations, approaches to epigenetic cancer therapy targeting TET are still limited. Our hypothesis is that combination treatment decreasing DNMT enzyme activity and increasing TET enzyme expression is expected to rapidly and effectively repair the silencing of tumor suppressor genes via aberrant hypermethylation in their CpG islands in cancer cells. To test this hypothesis, we prepared a new EpC nanocarrier that simultaneously delivers 5-aza-dC, a DNMT inhibitor, and TET1 gene-encoding pDNA to cancer cells and investigated its anti-tumor effects on HCT116 colon cancer cells. The results showed that EpC nanocarrier treatment successfully induces the expression of the major tumor suppressor gene TP53 in HCT116 cells. We also found that the co-delivery of 5-aza-dC and the pDNA of TET1 improved the response in epigenetic therapy against the cancer cells, leading to rapid cell cycle arrest in the G2/M phase. In addition, experiments suggested that EpC nanocarriers induce growth arrest rather than apoptosis induction in HCT116 cells.

Recent investigations have shown that the decreased expression of TET enzymes, mutations in TET genes, and lower 5hmC levels are general hallmarks of cancers [18]. However, TET mutations are uncommon in solid tumors and are frequently missense mutations that would not significantly impact TET activity [13]. In contrast, the 5hmC levels are significantly reduced in solid tumors, which is strongly associated with the downregulation of TET expression [19]. Since the oxidization of 5mC to 5hmC, the first step of the active DNA demethylation process, is catalyzed by TET enzymes, the activation or increased expression of TET enzymes should contribute to changing the phenotype of the cancer cells. In line with this scenario, several pharmacological TET enzyme activators have been reported recently. For instance, Sajadian et al. demonstrated that a combination of 5-azacytidine and vitamin C, an essential cofactor of TET enzymes, enhanced TET activity, leading to an increase in p21 protein and cell cycle arrest in hepatocellular carcinoma cells by the downregulation of Snail accompanied by GADD45B induction [20]. Kim et al. identified mitoxantrone as a potent TET agonist that induced tumor cell death by increasing 5hmC levels via the restoration of TET activity [21]. However, these activators upregulate the activity of TET enzymes in an expression-independent manner but do not directly amplify the expression of TET enzymes [22,23]. Therefore, in the present study, we evaluated the effects of the expression of TET enzymes on cancer treatment.

The results showed that the transfection of TET1 gene supports the effect of the DNMT inhibitor and accelerates G2/M cell cycle arrest. The activation of wild-type p53 triggers the cell cycle arrest by inducting the transcriptional downregulation of many genes that cause the cell division cycle to progress [24]. Therefore, after the fast promotion of the active DNA demethylation process catalyzed by the TET1 enzyme, the sustainable release of the DNMT inhibitor from the PLGA core nanoparticle of the EpC nanocarrier may contribute to maintaining the p53 level in the treated cells by maintaining low DNMT expression levels, leading to continuous cell cycle arrest.

Colon cancer is one of the most susceptible cancers to aberrant DNA methylation, and both TET1 expression and 5hmC levels are downregulated [25,26]. Among the TET family (TET1, TET2, and TET3), the suppression of TET1 expression is characteristic of colon cancer. In addition, the promoter CpG island of the TET1 gene is frequently methylated in multiple cancers [27]. The downregulation of TET1 is linked to cancer development and malignancy due to the epigenetic inactivation of the Wnt signaling pathway [28]. Furthermore, the depletion of TET1 in colon cancer cells is associated with the attenuated inhibitory effects of 5-aza-dC for DNMTs [29]. On the other hand, a significant reduction in 5hmC is observed in ca. 70% of colon cancer cases [30]. 5hmC is not just as an intermediate of the active DNA demethylation process but plays a role as an epigenetic modifier for the passive DNA demethylation process [31,32]. DNMT1 methylates the unmethylated strand of the hemi-methylated CpG sequence in DNA replication but does not recognize hemi-hydroxymethylated DNA [33]. Consequently, the maintenance DNA methyltransferase reaction of DNMT1 is suppressed. Therefore, the upregulation of TET1 in HCT116 colon cancer cells not only suppresses their proliferation but also may improve drug resistance to 5-aza-dC in our system, and the role of the DMNT inhibitor may involve maintaining the expression level of TET1.

These results suggest that the dual-targeting EpC nanocarrier could potentially serve as a new cancer therapy that differs from conventional anticancer drugs. However, this research is still in its early stages, and further validations, including the elucidation of the detailed mechanism of action of the EpC nanocarrier, are needed for its application in cancer therapeutic drugs. First, our findings are currently limited to in vitro experiments. The lack of in vivo experiments creates a significant gap because the tumor microenvironment and immune interactions may influence the epigenetic landscape and therapeutic response [34,35]. Future research should address this limitation by validating the functional relevance and therapeutic potential of the EpC nanocarrier using well-characterized xenograft models or other in vivo systems, together with comprehensive investigations of the underlying impact of the EpC nanocarrier on signaling pathways in tumor cells, with a particular focus on how the synergy between DNMT inhibition and TET activation regulates cancer-related upstream and downstream pathways at the molecular level. These efforts are essential in translating our findings from basic research into meaningful impacts in cancer treatment.

Another critical challenge is ensuring the efficient delivery of EpC nanocarriers to treat target tumors and uptake by cancer cells. The biological barriers within the human body can result in the significant clearance of nanocarriers before they reach the tumor site. Even after reaching the tumor, achieving optimal cellular uptake remains a hurdle due to the heterogeneity of cancer cells and their microenvironments [36,37]. Thus, precise bioengineering techniques, including the surface modification of nanocarriers with targeting ligands or employing stimulus-responsive release mechanisms, are required to design advanced nanocarriers that not only effectively encapsulate therapeutic agents but also specifically target cancer cells with the controlled release of encapsulated therapeutics while maintaining stability in systemic circulation and avoiding side-effects impacting other, normal tissues and cells. However, their implementation adds another layer of complexity to the design process. Developing such advanced nanocarriers into clinically usable drug delivery systems still poses technical and economic challenges. First, the manufacturing process of nanocarriers incorporating multiple drugs is often costly because it requires specialized equipment and sequential manufacturing lines, strict quality control, and scalability issues. Furthermore, because cancer is characterized by heterogeneity, customizing nanocarriers for different cancer types would further increase the complexity and manufacturing costs. However, core–shell-type lipid–polymer hybrid nanocarriers may overcome the cost-related challenges of the manufacturing process because they allow for easy tuning of the drug encapsulation rate and the size of the core nanoparticles, as well as high particle stability [38]. In addition, our approach to improving epigenetic abnormalities by restoring the balance of related enzyme activities may create universal nanocarriers independent of cancer type and mutation, while also overcoming cost-related challenges.

On the other hand, despite the promise of targeting epigenetic pathways, long-term safety remains a clinical concern in therapeutic applications [39]. Manipulating epigenetic mechanisms could potentially lead to altered gene expression in unintended ways and cause unforeseen consequences. In addition, acute and subchronic toxicity evaluations in various organs are needed for future clinical applications. Although these risks necessitate a rigorous evaluation of the safety profile of EpC nanocarriers in preclinical testing, such risk assessments will guarantee the safety of EpC nanocarriers in future therapeutic applications.

## 4. Materials and Methods

### 4.1. Cell Culture

HCT116 cells and WI-38 cells were obtained from RIKEN BRC Cell Bank (Tsukuba, Japan) and cultured in DMEM (Gibco, San Francisco, CA, USA) containing 10% FBS with 100 U/mL penicillin and 100 µg/mL streptomycin (FUJIFILM Wako, Osaka, Japan) at 37 °C with 5% CO_2_.

### 4.2. Preparation of EpC Nanocarrier

To a solution of 40 mg of PLGA (Sigma-Aldrich, St. Louis, MO, USA) in 2 mL of DCM/MeOH (3:2), a solution of 2 mg of 5-aza-dC (TCI, Tokyo, Japan) in 0.2 mL of distilled water/DMSO (3:2) was added dropwise and then sonicated for 1 min to form a W/O emulsion. The W/O emulsion was added dropwise to a 17.5 mL of PVA (Sigma-Aldrich) solution in distilled water (1 mg/mL) and then sonicated for 1 min to form a W/O/W emulsion. After organic solvents were removed by evaporation at 45 °C, the resulting emulsion was centrifuged at 12,500 rpm for 20 min. After the supernatant was discarded, distilled water was added to the emulsion, and the dispersion was freeze-dried. The inhibitor encapsulation rate was 27.1%, determined by the UV absorption of a DMSO solution of the PLGA particles.

Solutions (0.1 mL) of DOPE (TCI) and DOTMA (NOF corporation, Tokyo, Japan) in CHCl_3_ (10 mg/mL) and 2 mL of CHCl_3_ were placed into a flask, and the solvent was removed by evaporation to form a lipid thin film on the inner wall of the flask. Then, 1 mL of the 5-aza-dC-encapsulated PLGA particle dispersion (1 mg/1 mL) and 2 mL of PBS(−) were added to the flask, and the mixture was sonicated for 30 min to form lipid-coated PLGA nanoparticles. After incubation for 1 h, a solution of TET1-encoding pDNA (Addgene, Watertown, MA, USA) was added to the PLGA particle dispersion at varied N/P ratios, then the mixture was incubated for another 1 h to complete hybridization.

The mean diameters and zeta potentials of PLGA core nanoparticles, lipid-coated PLGA nanoparticles, and EpC nanocarriers in PBS buffer were measured with an ELSZ2 Zeta-Potential and Particle Size Analyzer (Otsuka Electronics, Osaka, Japan).

### 4.3. Agarose Gel Electrophoresis

Agarose gel was prepared by dissolving 0.6 g of UltraPure Agarose (Thermo Fisher Scientific, Cleveland, OH, USA) in 60 mL of TAE buffer (FUJIFILM Wako). Ethidium bromide (EtBr) (TCI) was added to the agarose gel solution. A gel tray was filled with running buffer. After loading a mixture of lipid-coated PLGA nanoparticles and pDNA at varied N/P ratios with a BlueJuice Gel Loading Buffer (Invitrogen, Carlsbad, CA, USA), electrophoresis was performed at a constant current for 30 min. Then, the fluorescence of EtBr intercalated into DNA was observed with a Gel Doc XR+ gel documentation system (Bio-Rad Laboratories, Hercules, CA, USA).

### 4.4. Luciferase Assay

HCT116 cells were seeded on a 96-well plate at 1 × 10^5^ cells/well and incubated for 24 h. Then, a mixture of lipid-coated PLGA nanoparticles and luciferase-encoding pDNA at varied N/P ratios was added to the cells, and they were incubated for 48 h. The cells were washed with PBS(−), then 20 μL/well of cell lysis buffer (5X) was added, and the luciferase activity of the lysates was measured with a Luciferase Assay System (Promega, Madison, WI, USA) according to the manufacturer’s protocol.

### 4.5. Alamar Blue Assay

WI-38 cells were seeded on a 96-well plate at 1 × 10^5^ cells/well and incubated for 24 h. Then, a dispersion of the EpC nanocarrier was added and incubated for a further 24 h. After the cells were washed with PBS(−), a fresh medium was added and incubated for 48 h. Subsequently, 50 μL of Alamar Blue Cell Viability Reagent (Invitrogen) was added to each well and incubated for 4 h. The cell viability was evaluated by absorbance with a DTX800 Multimode Detector (Beckman Coulter, Indianapolis, IN, USA).

### 4.6. Intracellular Localization

HCT116 cells were seeded on an 8-well Nunc Lab-Tek II Chamber System (Thermo Fisher Scientific, Waltham, MA, USA) at 5 × 10^4^ cells/well and incubated for 24 h. Then, lipid-coated PLGA nanoparticles encapsulating Rho-B dye (TCI) were added to the cells and incubated for a further 24 h. After washing with PBS(−), the cells were fixed with 4% paraformaldehyde and then counterstained with DAPI (FUJIFILM Wako) for nucleus visualization. The intracellular uptake of the nanoparticles was observed with a FLUOVIEW FV-10i confocal laser scanning microscope (Olympus, Tokyo, Japan).

### 4.7. Western Blotting

HCT116 cells were seeded on a 12-well plate at 1 × 10^5^ cells/well and incubated for 24 h. Then, the cells were treated with EpC nanocarriers and incubated for 24 h. After the cells were washed with PBS(−), 20 μL/well of cell lysis buffer (5X) was added. Proteins were separated by SDS-PAGE and then transferred to a PVDF membrane. The membrane was blocked with 5% non-fat milk and incubated with primary antibodies against p53 and β-actin antibody (Cell Signaling Technology, Beverly, MA, USA), and then secondary antibodies. Finally, the bands of p53 and β-actin were visualized with an ECL Prime Western Blotting Detection Reagent (GE Healthcare Japan, Tokyo, Japan). The chemiluminescence of the bands was detected with an AE-9300 Ez-Capture MG image analyzer (ATTO, Tokyo, Japan). The expression levels were quantified by ImageJ 1.54g (National Institutes of Health, Bethesda, MD, USA).

### 4.8. Cell Cycle Analysis

HCT116 cells were seeded on a 12-well plate at a concentration of 1 × 10^5^ cells/well. After 24 h of incubation, EpC nanocarriers were added and incubated for 48–120 h. Then, the cells were detached with trypsin and washed with PBS(−). Ethanol was added and incubated at 4 °C for 2 h. After being centrifuged at 5000 rpm for 10 min, the supernatant was removed and the cells were washed with PBS(−). After removing the supernatant, 1 mL of RNase A solution (Sigma-Aldrich) (0.25 mg/mL) was added to the cells, and they were incubated at 37 °C for 45 min. Then, the cells were stained with 50 µL of propidium iodide (PI) (50 µg/mL) at 4 °C for 30 min. The cell cycle was measured and analyzed with a CytoFLEX flow cytometer (Beckman Coulter).

### 4.9. Apoptosis Assay

HCT116 cells were seeded on a 12-well plate at a concentration of 1 × 10^5^ cells/well. After 24 h of incubation, EpC nanocarriers were added and incubated for 48 h. Cells were treated with trypsin and incubated at 37 °C for 10 min. Then, a fresh medium was added. After being centrifuged at 5000 rpm for 5 min, the supernatant was removed and the cells were washed with PBS(−). The apoptosis cells were detected using an Annexin V-FITC Apoptosis Detection Kit (Funakoshi, Tokyo, Japan) according to the manufacturer’s protocol. Briefly, the cells were suspended in 1 mL of binding buffer (1x) and labeled with FITC-Annexin V and PI and then incubated for 15 min in the dark. The fluorescence of FITC-Annexin V and PI was immediately detected with a CytoFLEX flow cytometer (Beckman Coulter).

### 4.10. Statistics

Student’s *t*-test was used to compare the difference between the two groups. A *p*-value of <0.05 was considered statistically significant.

## 5. Conclusions

In conclusion, as a method for performing DNA demethylation sustainably and more efficiently, we prepared a new epigenetics control (EpC) nanocarrier that simultaneously delivers a DNMT inhibitor, 5-aza-dC, and the pDNA of the TET1 protein, an enzyme for promoting DNA demethylation. The co-delivery of 5-aza-dC and TET1-encoding pDNA improved the response for the DNMT inhibitor, leading to rapid G2/M cell cycle arrest via the activation of p53 and TET1 expression in colon cancer cells.

The novel epigenetic therapeutic approach proposed in this study, which involves the inhibition of DNA methyltransferase activity and the induction of DNA demethylase expression in cancer cells, may be a useful concept for a future novel epigenetic cancer therapy method, different from conventional methods.

## Data Availability

The data that support the findings of this study are available from the corresponding author upon reasonable request.

## Supplementary Materials

The following supporting information can be downloaded at: https://www.mdpi.com/article/10.3390/epigenomes9010006/s1, Figure S1: Cell viability of HCT116 cells with various treatments. Figure S2: Flow cytometry analysis for the apoptosis in HCT116 cells (the data results from an independent experiment shown in Figure 6).
