# Supplementary material for: Novel Epigenetics Control (EpC) Nanocarrier for Cancer Therapy Through Dual-Targeting Approach to DNA Methyltransferase and Ten-Eleven Translocation Enzymes
Source: Epigenomes. 2025 Feb 11;9(1):6. doi: 10.3390/epigenomes9010006 (PMC11843842; doi:10.3390/epigenomes9010006)
Supplement: Supplementary file 1 [file epigenomes-09-00006-s001.zip › epigenomes-3379198-supplementary.pdf]

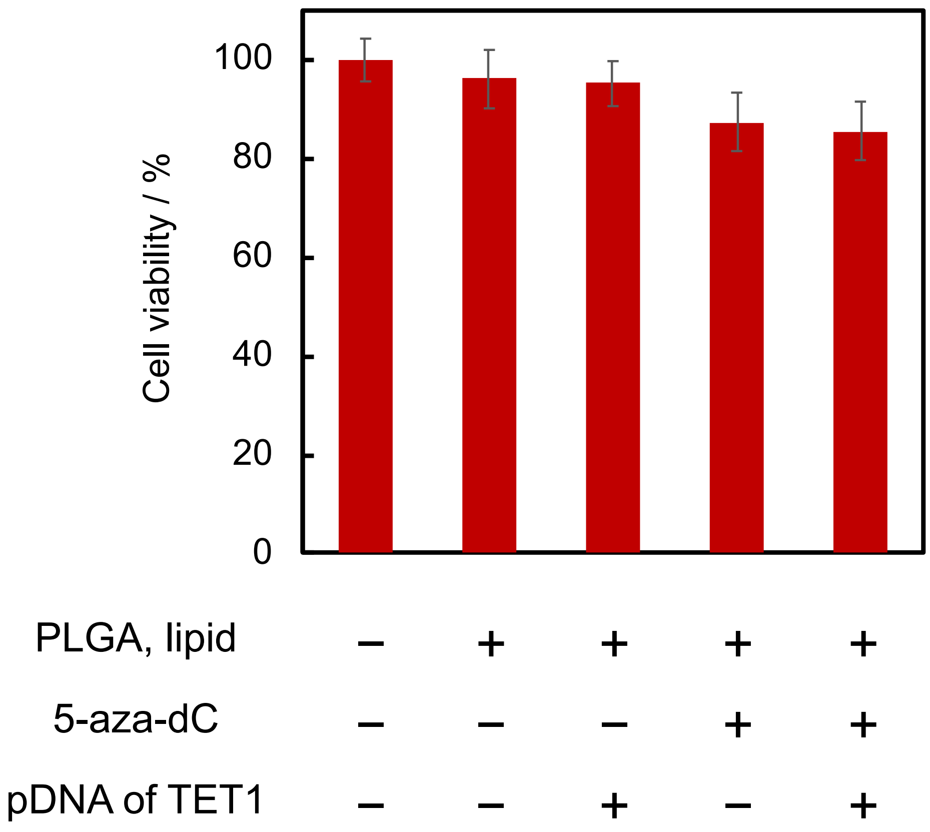

Figure S1. Cell viability of HCT116 cells with various treatments.

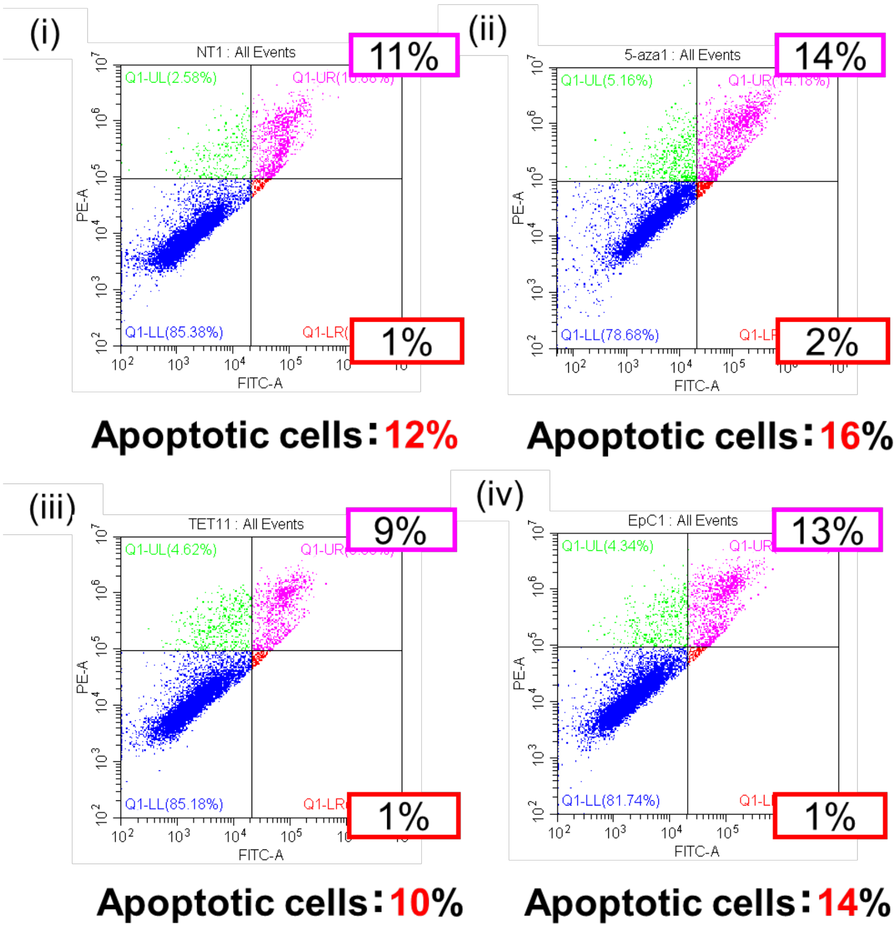

Figure S2. Flow cytometry analysis for the apoptosis in HCT116 cells (the data results from an independent experiment shown in **Figure 6**).

(i) NT, (ii) 5-aza-dC, (iii) TET1 carrier, and (iv) EpC nanocarrier.
